# Factors That Influence Enrolment and Retention in Ghana’ National Health Insurance Scheme

**DOI:** 10.15171/ijhpm.2017.117

**Published:** 2017-10-17

**Authors:** Agnes Millicent Kotoh, Genevieve Cecilia Aryeetey, Sjaak Van der Geest

**Affiliations:** ^1^School of Public Health, University of Ghana, Legon, Ghana.; ^2^Department of Sociology and Anthropology, University of Amsterdam, Amsterdam, The Netherlands.

**Keywords:** National Health Insurance (NHI), Enrolment, Retention, Drugs, Ghana

## Abstract

**Background:** The government of Ghana introduced the National Health Insurance Scheme (NHIS) in 2004 with the goal of achieving universal coverage within 5 years. Evidence, however, shows that expanding NHIS coverage and especially retaining members have remained a challenge. A multilevel perspective was employed as a conceptual framework and methodological tool to examine why enrolment and retention in the NHIS remains low.

**Methods:** A household survey was conducted after 20 months educational and promotional activities aimed at improving enrolment and retention rates in 15 communities in the Central and Eastern Regions (ERs) of Ghana. Observation, indepth interviews and informal conversations were used to collect qualitative data. Forty key informants (community members, health providers and district health insurance schemes’ [DHISs] staff) purposely selected from two casestudy communities in the Central Region (CR) were interviewed. Several community members, health providers and DHISs’ staff were also engaged in informal conversations in the other five communities in the region. Also, four staff of the Ministry of Health (MoH), Ghana Health Service (GHS) and National Health Insurance Authority (NHIA) were engaged in in-depth interviews. Descriptive statistics was used to analyse quantitative data. Qualitative data was analysed using thematic content analysis.

**Results:** The results show that factors that influence enrolment and retention in the NHIS are multi-dimensional and cut across all stakeholders. People enrolled and renewed their membership because of NHIS’ benefits and health providers’ positive behaviour. Barriers to enrolment and retention included: poverty, traditional risk-sharing arrangements influence people to enrol or renew their membership only when they need healthcare, dissatisfaction about health providers’ behaviour and service delivery challenges.

**Conclusion:** Given the multi-dimensional nature of barriers to enrolment and retention, we suggest that the NHIA should engage DHISs, health providers and other stakeholders to develop and implement intervention activities to eliminate corruption, shortage of drugs in health facilities and enforce the compulsory enrolment stated in the NHIS policy to move the scheme towards universal coverage.

## Background

Over the last two decades many developing countries have been experimenting with social health insurance schemes (SHISs) to improve healthcare access. In Ghana, the National Health Insurance Scheme (NHIS) was introduced in 2004 in response to the criticism that user-fees,^ [1]^ particularly cash and carry,^ [2]^ improved the quality of care and drug supply but denied many people access, especially vulnerable groups.^1-4^

This criticism led many organisations including International Labour Organisation (ILO) and the World Health Organization (WHO) proposing the establishment of a national health insurance (NHI) to improve healthcare access in Ghana. The Provisional National Defence Council (PNDC) responded to this suggestion by contracting experts to make recommendations for creating a NHI organisation. They proposed the establishment of a centralised company to provide a compulsory social health insurance for all Social Security and National Insurance Trust (SSNIT)^ [3]^ contributors and registered cocoa farmers. Rural-based community-finance schemes were also proposed for non-formal sector workers. These proposals culminated in the establishment of community-based health insurance schemes (CBHISs) in the early 1990s with support of the Ministry of Health (MoH) and international donors. The National Democratic Congress (NDC) government also launched a pilot NHIS in four districts in Eastern Region (ER) in 1997. This scheme, though stalled, stimulated debate on the need to find an alternative sustainable healthcare financing system.^5^ The government, inspired by the modest success of some CBHISs continued the initiative of establishing a viable NHIS.^5-7^ But this vision was curtailed by a change in government in January 2001.

The main opposition party, New Patriotic Party (NPP), having made a commitment during the 2000 election campaign to implement the NHI, initiated the policy-making process. Consequently, the NHI Law (Act 650) was passed in 2003^8^ and district health insurance schemes (DHISs) were established in all districts in the country. The NHIS became operational in March 2004 to replace cash-and-carry and ensure equity in healthcare access. The NHIS’ goal is: “*Within the next five years, every resident in Ghana shall belong to a health insurance scheme that adequately covers him or her against out-of-pocket payment ….”*^9,10^ The NHI Act specifies the specific diseases and healthcare services covered and not covered.^11,12^ However, the insured could only access healthcare in accredited facilities within the district where they registered.

The NHIS premium is subsidised by 2.5% value added tax (VAT). The SSNIT contributors pay 2.5% of their contributions as premium (Table 1). Non-SSNIT contributors are expected to pay an income adjusted premium of between GH₵22 (about US$10) and GH₵48 (about US$22) per adult per annum; but in practice everybody pays the minimum. Exemptions are provided for vulnerable: children below 18 years, the elderly (70 years and above) and the core poor. Pregnant women and mentally challenged persons were added to the exemption category in 2008 and 2012 respectively.^13,14^

**Table 1:** Current Features and Operational Principles of the NHIS

**Features**	**Description**
Administration	- DHISs are centrally administered by the NHIA but day-to-day administration is decentralised to the districts. - NHIA functions as the insurer; provides NHIS cards and accreditation to service providers, negotiates benefit packages, cost of care, ensures quality service and pays service providers.
Funding	- 2.5% VAT. - 2.5% SSNIT contribution. - Money allocated to the NHIF by Parliament. - Income from investments. - Premium from non-SSNIT contributors, registration and administrative fees. - Donations from non-governmental organisation and individuals.
Membership	- Membership is compulsory for all residents in Ghana; unless private health insurance membership can be shown .
Exemptions	- SSNIT pensioners, non-SSNIT contributors above 70 years, children below 18 years, pregnant women and mentally challenged persons.
Benefit package	- Covers 95% of diseases reported in health facilities in Ghana.
Payment to service providers	- Pay service providers within four weeks of claim submission to DHISs.
Supervision	- The NHIA regulates premium and registration fees. - Health facilities submit quarterly reports to the NHIA. - DHISs submit annual reports to the NHIA who audits their accounts.

Abbreviations: DHISs‏, district health insurance schemes; NHIA, National Health Insurance Authority; VAT, value added tax; SSNIT, Social Security and National Insurance Trust; NHIF, National Health Insurance Fund; NHIS, National Health Insurance Scheme; NHIF, National Health Insurance Fund.

The NHIS, the first nation-wide scheme in Africa initiated by a government, covers both formal and informal sector workers and operates under a nationalised system of service provision and financing with no co-payment. Healthcare is obtained from all public health, faith-based, quasi-government and some private health facilities, pharmacy and chemist shops that have been accredited and operate under contract with the National Health Insurance Authority (NHIA).

The NHIA established and manages the National Health Insurance Fund (NHIF). Premium payment account for 5%, SSNIT 17.5%, VAT 73% and interest on investment 5.2% of NHIF’ inflows.^15^ The NHIS has become the dominant source of income for public health facilities; accounting for 79.4% of internally generated funds (IGF) in 2010.^16^ and reduces out-of-pocket health expenditures (OOPHEs).^17^

Ghana is a lower middle income country with estimated gross domestic product (GDP) of US$38 617 billion.^18^ Tax constitute 14.9% of GDP in 2011.^19^ The proportion of GDP spent on health in 2014 wa 3.6%.^18^ The per capita expenditure on health was US$145.^18^ Per the 2010 Population and Housing Census, 71.1% of the population aged 15 years and above were economically active.^20^ The poverty incidence (PI) in 2012 was 24.2% and poverty gap index 7.8%.^21^

Access to healthcare has increased through the establishment of community-based health planning and services (CHPS)^ [4]^ zones nation-wide. However, human resource challenges persist. With the ratio of one doctor to 10 402 and one nurse to 1599 persons in 2011,^22^ and other service delivery challenges, quality service is often comprised^23^ and the country continuous to experience poor health indicators. Infant mortality rate was 41 deaths per 1000 live births, under five mortality 60 deaths per 1000 live births in 2014^24^ and maternal deaths (380 per 100 000 life births) in 2013.^22^ Life expectancy at birth in 2014 was 60.3 years and 62.5 year for males and females respectively. The literacy rate was 74.1%.^20^

Though studies have consistently shown that NHIS provides quick access to healthcare,^25,26^ only 34% of the population was enrolled in 2011.^27^ In the light of the relatively low NHIS premium and exemptions for vulnerable groups, factors that undermine enrolment and retention in the scheme need to be explored.

Previous studies attribute low enrolment in SHISs to poverty, inadequate information about health insurance and perceived poor quality of service.^28-31^ These researchers who often focused on one stakeholder, usually community members and used only quantitative methods, gain limited information about the phenomenon of low enrolment in SHISs. This study examined factors that influence enrolment and retention in the NHIS using a multi-level perspective (MLP) which emphasises engagement of stakeholders at different levels in studying healthcare^32-34^ to more convincing explanation to the phenomenon of low enrolment and high drop-out rate.

The word ‘level,’ a metaphor, refers to the international, national, regional and local tiers of social organisation.^33^ Engaging actors and focusing on events at these levels is the objective of the MLP. The authors emphasised that the actors’ varied interest and events interact to affect a social phenomenon. We thus propose that in order to draw more convincing conclusions about why enrolment is low and retaining members in the NHIS a challenge, it is necessary to focus on both local and national stakeholders of the scheme, namely: community members, health providers, and staff of DHISs, the NHIA, Ghana Health Service (GHS) and MoH and use ethnographic tools in the study. The findings of this study will not only inform policy and intervention activities to move NHIS towards universal coverage, but also help other developing countries address problems confronting their SHISs.

## Methods

### Study Sites

This ethnographic study was conducted in the Central Region (CR) and ER of Ghana. The two regions were strategically chosen to reflect the ecology (forest and coastal) and the main economic activity (agriculture) of Southern Ghana. The CR and ER had 70% and 72.7% economically active population aged 15 years and above.^20^ Unemployment rate was 8.1% and 8.4%^21^ and poverty incidence (PI) 18.8% and 21.2% in the CR and ER respectively.^21^ The literacy rate in the CR and ER were 81% and 78.2% respectively.^20^

Each region has a regional hospital. All districts used in this study have a hospital, health centres, clinics and CHPS zones. Almost all communities fall within the 5-km radius proximity to a health facility.

Some key health indicactors in the two regions for 2011 were: infant mortality (per 1000 live births) was 61 and 50, maternal mortality (deaths/100 000 live births) 520 and 538, and total fertility rate was 3.6% in the CR and ER respectively.^21^

### Selection of Participants and Quantitative Data Collection

Since a health economist on the research project has discussed the sample size calculation in detail elsewhere,^35^ we limit ourselves to giving the sample size and the selection process, and discuss the qualitative method in this paper. The sample size of 3000 households for the whole project was based on 80% power to detect a 5% difference in overall enrolment between intervention and control communities and 10% was added to cater for non-response rate; making the total sample 3300 households (110 for each community).^35^

The survey sample was selected as follows. First, one census enumeration area (EA) referred to as community in this paper was randomly selected from each of the 30 districts with DHIS offices using computer generated random numbers. Second, all households were listed and 110 randomly selected.

A structured questionnaire was administered to heads of households. The survey data was used to categorise the communities into 15 interventions and 15 controls; considering their socio-economic status (SES), NHIS status and rural/urban location.

This paper is based on the follow-up survey carried out in March 2011 after exposing the intervention communities to 20 monthly educational and promotional activities to ensure people have adequate knowledge of NHIS’s operational principles and benefit package. Qualitative data was collected between June 2009 and September 2011. The details of intervention activities are reported elsewhere.^36^

### Quantitative Data Analysis

We used crosstabs to analyse enrolment status of individual household members, reasons for enrolling, not renewing membership and never enrolling. Chi-square test was used to determine the relationship between NHIS status, socio-economic and perceived health status.

The health economists estimated the PI using household detailed monthly consumption expenditure on food and non-food items and dwelling characteristics (eg, water supply and availability of electricity etc).^37,38^ These indicators conform to the definitions of poverty in the Ghana Living Standard Survey (GLSS) 6 data.^21^ The GLSS is an internationally accepted method for estimating PI in developing countries. The estimated monthly household expenditure represented the total amount of money needed to meet food and non-food consumption requirements of households.^21^ These estimates were considered as proxies for household wealth. Principal component analysis, a statistical procedure used to determine weights for a linear index of a set of variables was employed to estimate households’ SES scores.^39,40^ The households were ranked into five wealth quintiles based on their SES scores (core poor, poor, average, rich and very rich).^37,38^ All the analysis was done in SPSS version 16.

### Key Informants and Qualitative Data Collection

Qualitative data was collected in seven communities in the CR by the first author using observation, in-depth interviews and informal conversations. She paid special attention to two strategically selected communities: an urban fishing community and a rural farming community in the forest zone and their respective health facilities and DHIS offices as case studies (Table 2).

**Table 2:** Profile of Key Informants in Each Case Study Community and National Level

	**Position/Employment**	**Number**
**Local Stakeholders (n = 20)**
Community members	Fishermen or farmers	3
	Traders/farmers	2
	Unemployed	1
	Traditional and Opinion Leaders	3
	Religious Leaders	2
Health facility staff	Staff Nurse	1
	Community Health Nurse	1
	Midwife	1
	Medical Assistant	1
	Physician	1
‘District Health Directorate’ staff	Disease Control Officer	1
	DDHSs	1
DHIS staff	Public Relations Officer	1
	Manager	1
**National Level Stakeholders (n = 4)**		
MoH	1	
GHS	1	
NHIA	2	

Abbreviations: DHIS, District Health Insurance Scheme; MoH, Ministry of Health; GHS, Ghana Health Service; NHIA, National Health Insurance Authority; DDHSs, District Director of Health Services.

Forty local key informants were interviewed (20 from each case study site: 11 community members, 7 health providers and 2 DHIS’ staff). In addition, four national level key informants (one each from the MoH and GHS headquarters and two from the NHIA) were interviewed. The community members were cautiously selected to ensure they fairly represent the target population: currently insured, previously insured and never insured. Educational level, SES and health status were also considered. Key informants who were health providers, staff of DHISs, GHS, NHIA and MoH were purposely selected based on their work schedule.

Several community members, health providers and DHISs’ staff who were not regarded as key informants were engaged in informal conversation in the seven communities in the CR. Informal conversation, unstructured spontaneous discussions with participants, provides the opportunity to ask pertinent questions on different occasions. This eliminates the possibility of participants adjusting their response on purpose or holding back information on sensitive issues that are critical to the study.

### Qualitative Data Analysis

Observations and conversations were jotted down and elaborated into field note books at the end of each day in line with standard ethnographic studies.^41^ Second, digitally recorded interviews were transcribed verbatim and subjected to content analysis to elicit the common themes emanating from the data. Third, typical comments by participants were condensed into meaningful summary statements and placed under the appropriate theme. Fourth, all authors reviewed the analysis to ensure that the themes and summary statements reflect the participants’ views.

The data was collected at multiple levels using both quantitative and qualitative methods in order to capture what happens at various levels and uncover aspects of issues that are not immediately obvious.^42^ In this study, triangulation was used to verify responses by asking different categories of participants the same questions using interviews and informal conversation to capture all dimensions of factors that influence health insurance decisions.

Four main steps were taken to ensure validity and authenticity of the data collected. (1) The questionnaire and interview guide were translated using the back-translation method and pre-tested. (2) The first author conducted all interviews. (3) Triangulation was used to verify responses. (4) Self-reflection, etic^ [5]^ and emic^ [6]^ perspectives were used to ensure objectivity when writing the reports.

## Results and Discussion

### Background Characteristics of Households and Individuals Covered in the Survey

Of the 6790 individuals covered by the survey, 46.3% were under 18 years and 4% were 70 years and above. Females formed 52.5% (Table 3). These statistics roughly agree with the 2010 Population and Housing Census which reports that 38.3% and 4.7% of the population were under 15 years and 65 years and above respectively.^20^

**Table 3:** Background Characteristics of Households and Individuals Covered in the Survey

**Overall (All Individuals), N = 6790**	**Percent**
Age, n = 6790	
0-17	46.3
18-69	49.7
70+	4.0
Gender, n = 6790	
Male	47.5
Female	52.5
Highest level of education of heads of households, n =1562	
None	26.3
Primary	20.6
Secondary	44.4
Tertiary	8.7
Marital status of heads of households, n = 1562	
Single	9.0
Married	62.0
Divorce	6.8
Separated	5.5
Widowed	12.5
Cohabiting	4.2

### Enrolment in the National Health Insurance Scheme and Membership Renewal

Community members typically described the NHIS as: “*A good arrangement that ensures everyone has access to healthcare*.” However, this did not translate into high enrolment and regular renewal of membership. Of the 6790 individuals covered by the survey, 40.3% were currently insured and 22.4% previously enrolled.

Significant differences were observed between enrolment of poor and rich respondents as well as the sick and healthy. Lower enrolment was reported among the poor categories. Of the 1392 poorest individuals covered, 17.6% were currently insured compared to 44.4% out of the 1299 richest (*P* = .000). Membership non-renewal rate was lower among the poor: poorest (15.4%) and richest (23.8%) (*P* = .000).

Respondents with perceived poor health enrol and renew their membership compared to healthy respondents (*P* = .000). Out of the 145 respondents perceived as having poor health, 73% were currently insured and 10.8% previously insured while 39.2% of the 6206 respondents with perceived good health were currently insured and 22.9% previously insured (Table 4).

**Table 4:** NHIS Status by Socio-Economic and Perceived Health Status

		**Currently Insured**	** Previously Insured**	**Never Insured**	***P***
Overall	N = 6790	40.3	22.4	37.3	
Socio-economic categories	N = 6790				
Poorest	1392 (20.5)	17.6	15.4	67.0	.000
Poor	1362 (20.1)	31.3	18.4	50.3	
Average	1336 (19.7)	35.0	22.1	42.9	
Rich	1401 (20.6)	46.4	23.7	29.9	
Richest	1299 (19.1)	44.4	23.8	30.9	
Perceived health status	N = 6788				
Good health	6206 (92.9)	39.2	22.9	37.9	.000
Average health	339 (5.0)	56.3	14.2	29.5	
Poor health	145 (2.1)	73.0	10.8	16.2	

Abbreviation: NHIS, National Health Insurance Scheme.

These statistics and qualitative data revealed factors that influence enrolment and retention in the NHIS are multi-dimensional. The factors are discussed under two themes: enablers and barriers.

Enablers of Enrolment and Retention in the National Health Insurance Scheme

Enablers are factors that inspired people to enrol and remain in the NHIS. These are benefits derived from the NHIS and positive health provider-patient interaction.

### Benefits of the National Health Insurance Scheme

Community members mentioned that the NHIS gives access to healthcare and financial relief from catastrophic payments. An insured diabetic patient described NHIS’ benefits as: *“The premium compared to the cost of healthcare is reasonable. I don’t have to spend all my money paying hospital bills. Insurance helps me get my drugs. I don’t have crises anymore.”*

Health providers confirmed these assertions and added that they encouraged people to enrol because the NHIS reduces complications among insured patients. A physician described the NHIS’ benefits as: “*One good thing about NHIS is that more people report to the hospital early with fewer complications and come for review regularly*.”

Our survey results support these accounts and show that more than two-thirds of respondents across the five SES enrolled because the NHIS provides financial protection against ill health (Table 5). Our results corroborate the findings that the NHIS improved access to formal care,^25,26^ significantly reduced out-of-pocket payment (OOPP).^43^ Durairaj et al observed a decline in hospital deaths among insured patients owing to early treatment.^26^ These evidence underscore the importance of the NHIS as a safety net for residents in Ghana.

**Table 5:** Reasons for Enrolling, not Renewing Membership and Never Enrolling in the NHIS by SES

**N = 1562**	**Poorest**	** Poor**	**Average**	**Rich**	** Richest**
Reasons for enrolling in the NHIS, n = 619					
Financial protection against illness	71.0	79.1	77.5	74.1	72.4
It a better than cash and carry	29.0	20.9	21.1	25.4	26.5
The school insured my child	0	0	1.4	0	0.6
Community opinion leaders asked me to join	0	0	0	0	0.6
Employer paid	0	0	0	0.5	0
Reasons for not renewing membership in the NHIS, n = 319					
Could not afford renewal payment	75.0	63.2	65.5	56.6	65.2
Not satisfied with service	25.0	5.3	6.9	10.5	13.0
Difficulty in accessing services	0	5.3	3.4	5.3	4.3
No transport money	0	0	0	0	1.3
Inappropriate timing of premium payment	0	5.3	3.4	1.3	0
Had to buy drugs outside facility	0	0	10.5	2.6	6.9
Did not use service last year	0	21.0	10.3	18.4	6.6
Others	0	0	0	5.3	2.7
Reasons for never enrolling in the NHIS, n = 608					
Cannot afford premium	100	96.8	61.3	70.5	66.7
Covered elsewhere	0	0	0	2.3	3.7
Mostly healthy do not need to be ensured	0	0	16.1	12.4	13.1
No scheme in the area	0	0	0	0	1.7
No close facility in the area	0	0	0	1.1	0
No confidence in the scheme	0	3.2	12.9	10.3	3.7
Registration point too far	0	0	3.2	0	7.4
Others	0	0	6.5	3.4	3.7

Abbreviations: NHIS, National Health Insurance Scheme; SES, socio-economic status.

### Positive Health Provider-Patent Interaction

Insured patients mentioned that they enrolled and remained in the NHIS because of some health providers’ positive behaviour towards them. One of them shared his experience as follows: “*I registered because the physician assistant (PA) persuaded me to enrol. The first thing they ask you is whether you have insurance card.”* The PA confirmed this assertion and said: *“I show patients my insurance card and educate them about the benefits to encourage them to enrol and renew their membership regularly to enable them seek care early and avoid complications.”*

These comments indicate that when health providers behave favourably towards insured patients, people are encouraged to enrol. Thus, perceptions about quality of service ultimately influence people to enrol in SHISs.^5^

### Barriers to Enrolment and Retention in the NHIS at the Community Level

Barriers are factors that discourage people from enrolling in the NHIS and renewing their membership regularly. The appreciation of the NHIS’ benefits was not marked by high enrolment and retention rates. Aside poverty, which is often reported in health economics literature as the main cause of low enrolment,^28-31^ this study reveals more hidden factors. These are influence of traditional risk-sharing arrangements, corruption among health providers, service delivery challenges and politics.

### Poverty/“No Money to Pay Premium”

Though poverty contributes to low enrolment, it is only an important factor among the poorest and some poor households with many members. When community key informants were questioned about why enrolment and retention in the NHIS is low, ‘No money to pay premium’ was normally their first response. Our survey results confirmed the ‘No money to pay’ reason and show that 63.2% poor and 65.2% richest respondents did not renew their membership in the NHIS and 70.5% rich and 66.7% richest never enrolled because of ‘poverty’ (Table 5) but when I engaged key informants and others who by local standards were not very poor in informal conversation, they gave additional reasons: ‘I’m not often sick’ and “I’m waiting for a while.” Critical analysis of their living conditions revealed that some poor household heads could pay the heavily subsidised premium for all their members but did not. I also observed that in many of the households, only those who needed healthcare were registered and renewed their membership regularly. So, I engaged them in discussions to explore their motives. Many participants stated that apart from the core poor and poor households with many dependants, low enrolment and retention was an attitudinal issue and not a matter of poverty. An uninsured cocoa farmer said: “*It is sad that most of us can pay the premium but we don’t. I registered my children and wife because they need healthcare.*”

I also observed that heads of households knew that OOPP is more expensive but waited and rushed to enrol or renew their membership for only members who needed healthcare. Also, the higher non-renewal rates among the average and rich categories in this study contrasts health economics literature in sub-Sahara Africa, which gives excessive weight to poverty as the main factor for low coverage of health insurance schemes.^44,45^

Two explanations are here in place. First, ‘poverty’ is not only an issue of lack of money but also lack of control over one’s own life uncertainties. As a result, people wait until they need healthcare before enrolling. For example, the majority (65.2%) of the richest respondents said they could not renew their membership because of poverty. Secondly, the ‘no money to pay’ statement had little to do with lack of money. It was a socially acceptable response.

That said, a critical analysis of the situation of some poor household heads who can pay the heavily subsidised premium but did not, revealed that the issue was beyond the premium. The poor do not have substantial income but due to the social responsibility of caring for close relatives in Ghana, the poor (usually the men) also have many dependents (their wives, children and other dependents) to enrol. Given their low income, enrolling all these dependents is unaffordable to them. A cocoa farmer and a father of six children (one above 18 years) and two dependents above 18 years explained that he did not have money to pay for everybody so he enrolled his wife and four children who needed healthcare.

In addition, all the poor who were engaged in informal conversation complained that the extra payments for healthcare services and drugs discouraged them from renewing their membership. A fisherman said: ‘*We struggle to enrol but when we go to the hospital, we pay for drugs or are given prescription to look for the drugs in accredited pharmacy shops which we sometimes pay for. This discouraged many people from enrolling and renewing their membership.*’ This supports the observation that compulsory or voluntary informal payment is a barrier to healthcare access for poor families; about 25% of healthcare users in Ghana pay illegal fees to public health providers.^46^

Our results show that poverty as a barrier to enrolment and retention was most important only for the core poor. Some of them did menial jobs and did not have regular income. Others were totally unemployed and occasionally supported by family members and neighbours. The majority (75%) of previously enrolled and all never enrolled core poor said they could not afford the cost of premium (Table 6). Appraisal of the qualitative data revealed that some of them could not even enrol their sick household members or take them to the hospital. A health volunteer describes the situation of the core poor as:

**Table 6:** Opinion Related to Quality of Service

**N = 1562**	**Currently Insured (n = 619)**			**Previously Insured (n = 319)**			**Never Insured (n = 608)**		
	**Agree**	**Neutral**	** Disagree**	** Agree**	** Neutral**	** Disagree**	** Agree**	** Neutral**	** Disagree**
The insured still have to buy drugs	64.0	11.6	24.4	58.2	10.8	31.0	50.0	19.8	30.2
Attitude of health staff should be improved	76.6	14.7	8.7	87.0	9.6	3.4	74.7	18.3	7.0
Availability of drugs should be improved	83.7	11.2	5.1	90.1	7.1	2.8	78.6	17.7	3.7
Expect prompt treatment at the facility	75.8	11.4	12.8	68.8	18.5	12.7	64.7	21.6	13.8

*“Many of the core poor do not have a stable source of income. They are occasionally supported by family members and friends. Even to get one meal a day is a problem. They cannot afford the cost of premium. Meanwhile they are not given exemption.”*

A core poor woman explained why she could not enrol her household members as follows: “*I do menial jobs and have no money to enrol my five children. One of them died because I had no money to take her to the hospital.*” Our results thus provide credible evidence that the core poor, who need health insurance most, could genuinely not afford the premium and so need exemption. However, the exemption is not reaching them. None of the core poor I engaged in conversation in the seven communities visited benefitted from exemption. When I questioned DHIS staff why they do not grant exemption to the core poor, one of them said: “*We need money so if we go about saying we want people to exempt they won’t pay and how do we get money?”* Witter and Garshong also reported that only one per cent of the population were granted exemption in 2008.^47^ Our results corroborate WHO’s finding that exemption is crucial to ensure that the poor are enrolled in SHISs.^48^ But just like previous exemption policies which were not successful,^49-51^ the purpose of the NHIS as a safety net has failed to reach the poor and ensure that they have access to healthcare when sick.

### Negative Influence of Traditional Risk-Sharing Arrangements

The fact that people quickly enrolled or renewed their membership when sick, indicates that they accept the NHIS as better than OOPP, but devised strategies to derive maximum benefit with minimal contribution. Almost all non-SSNIT members including the richest pay the minimum premium. An uninsured cocoa farmer provided insight into the actual situation in the following comment: “*Most of us can enrol and renew our membership during harvest time but we do not. We wait until we are seriously sick and rush to pay the minimum premium.”* I also met people who enrolled because they needed healthcare. During one of my routine observational visits to a DHIS office, I met a man who looked desperate and asked him what was wrong. He responded angrily: “*Madam I thought I didn’t need health insurance till I fell sick. Now I have registered and need the card for surgery but its delaying*.”

Our survey results support these comments and show that those who perceive themselves as healthy enrol less and have higher drop-out rate: 39.2% with perceived good health were currently insured and 22.9% previously insured compared to 73% with poor health status being currently insured and 10.8% previously enrolled (*P* = .000) (Table 4). This corroborates Kusi and colleagues’ finding in their 2011 study in three districts across Ghana that 73.9% of household members whose perceived health status was poor were likely to be insured compared to 49.2% with excellent health.^31^

Also, the 2014 nation-wide Demographic and Health Survey (DHS) reported that 48% and 62% of men and women respectively were currently enrolled. It must however be noted that the DHS covers only women of reproductive age (15-49 years) who enjoy free enrolment for pregnant women. Therefore, the 62% enrolment for women is not representative of all women. The survey also reported that 1% of the respondents were covered by other types of insurance.^24^ Blanchet et al also found that less than 25% of women under 30 years and about 45% over the age of 60 were enrolled in the NHIS.^52^ These results indicate that low enrolment is a country-wide phenomenon. The question then is: Is it really the case that 65.2% of richest previously enrolled and 66.7% richest never enrolled respondents genuinely could not pay the heavily subsidised premium?

All key informants attributed low enrolment and high drop-out rate to the negative influence of traditional risk-sharing arrangements used to manage livelihood activities and life events. An analysis of the operations of two of such groups: ‘*Pataase’*^ [7]^ in fishing communities and ‘*Nnoboa’*^ [8]^ in farming communities shows that though they have health insurance elements, their risk-sharing principles are not the same. People join ‘*Nnoboa’* when they need support on their farms and pull out from the group until another farming season. Benefits are commensurate to one’s contribution and not need. ‘*Pataase’* focus on life events, mainly death. People join and remain in ‘*Pataase’* because they are sure of benefitting. The benefits cover the funeral cost of members, their spouse, children and parents. A member argued: “*Death is a certain event but for ill health, you may or may not fall sick.”* A community leader explained low enrolment in the NHIS and retention as follows:

*“Health insurance is not part of our culture. People join ‘Nnoboa’ if they need help in their farms. Benefits are according to one’s contribution. You remain a member only when there is work to be done.”*

These arguments show that though solidarity and reciprocity are predominant features of both traditional risk-sharing arrangements and health insurance, the former do not help convince people to join and remain in the NHIS when healthy. Platteau’s review of concepts underlying traditional risk-sharing reveal that traditional mutual support schemes are based on balanced reciprocity (people receive as much benefit as they contribute),^53^ while insurance is based on conditional reciprocity (members receive a return only if they fall sick). Our study shows that people’s reaction to health insurance is influenced by the principles of traditional risk-sharing arrangements. The logic of not enrolling was that people perceive NHIS’ benefits as limited to the individual so their investment might not benefit them. As contended, a well-established cultural perspective limits the possibilities for thinking and acting in new situations.^54^ Our study also shows that existing knowledge and practices largely determine one’s reaction to new policies and not simply its benefits. The incentive for enrolling in the NHIS is largely informed by the motive of benefitting and not of sharing cost so people enrol when sick and opt out when well. This undermine the fundamental principle of health insurance; regular contribution into a common fund based on income, whether one benefits or not.

### Accusation of Corruption Among Health Providers

Community members mentioned that health workers exploit insured patients. They cited illegal payments for drugs and other services.

#### Payment of Illegal Fees

Throughout the fieldwork, the issue of extra payments by insured patients was prevalent. During an informal conversation, an insured woman narrated her experience at a health facility as follows:

*“I went to the hospital in the evening because of a sudden stomach pain. The nurse refused to accept my insurance card because I was late and demanded cash. I left and bought drugs from the chemist shop. I can go and show you the nurse.”*

I could not follow it up, but I asked a nurse from the facility to react to the complaint. She replied: “*I won’t deny it. Some of us ask insured patients to pay cash because we don’t want to fill the complex insurance form*.” In another incident, a health volunteer called me to intervene and collect money an insured patient was forced to pay. I followed-up to have concrete evidence to support the earlier reports I had received. The provider gave ‘a face-saving’ explanation and quickly refunded the money.

### Payment for Drugs Inside and Outside Health Facilities

Paying for drugs was a common complaint among insured patients. A community member told me: “*I had to* pay for *malaria drugs even though I know it is wrong*.” Though some health providers denied these allegations, others confirmed the practice and lamented on how these undermined the NHIS’ credibility. One of them said: “S*ome of us sell drugs that are covered by the NHIS to insured patients, ‘pocket the money’ and charge the DHISs.”* A medical officer confirmed the allegations and said: “*One of my patients reported to the nurse that she was not given all her drugs but given prescription to look for it outside. We followed-up and found that it was true, yet the DHIS was billed.”*

To explore these allegations further, I asked DHIS officials for their reaction. One of them lamented: “*Collecting illegal fees and payment for drugs undermine our efforts. Some of the people we struggle to enrol do not renew their membership because of the extra payments*.” These assertions support the evidence that payment of unauthorised fees has been a problem in the health sector in Ghana.^23,55,56^ Our study revealed that some health providers only pursue their parochial interests and not the achievement of NHIS’ goals. Our findings thus illuminate the observation that corruption undermines achievement of public policy goals.^57^

## Service Delivery Challenges at the District Health Insurance Schemes

Inadequate office accommodation, equipment and materials undermined the efficient functioning of DHISs. They were unable to deliver NHIS cards to their clients promptly. It was common to see many clients waiting for hours at the DHIS offices to get their NHIS cards. A DHIS staff explained: “*Inadequate equipment and registration materials make it difficult for us to deliver the cards to clients promptly*.*”*

Community members on their part expressed worry about the delay in getting their cards. I engaged some people I met in DHISs’ offices looking frustrated in a conversation. One of them told me: “*This is the second time I’m coming here without getting my card. I continue to pay at the hospital because of the delay.”* This study thus shows that service delivery challenges do not only frustrate DHIS staff but also discourage people from enrolling and remaining in the NHIS.

The ongoing biometric registration to replace the old system is expected to introduce efficiency into the process but it seems not to solve the problem of delay in getting NHIS cards. Anecdotal reports and my observation reveal that people still wait in long queues to register and do not sometimes get the cards immediately as expected due to inadequate equipment and shortage of materials.

## Healthcare Service Delivery Challenges

The assumption that the NHIS will improve quality of service was not evident. Health providers’ heavy workload and shortage of drugs on the NHI Drug List at health facilities undermined the quality of service.

### Heavy Workload and Long Waiting Time

Many people, who had no access to formal care or cut their treatment short because of user-fees,^1-4^ have access now. Though an improvement in utilisation is desirable, it has increased health providers’ workload. We found that the NHIS was implemented within an overburdened health system without adequate resources to handle the growing patient numbers. Patients waiting for hours at health facilities was a common sight. Insured patients argued that they anticipate prompt treatment, but they were rather made to wait longer than the uninsured. Their common complaint at busy health facilities was: “*Health providers make us wait while they attend to those who pay cash.”* Our survey results confirmed these assertions. The majority (75.8%) currently insured and (68.8%) previously insured respondents said they expect prompt treatment at health facilities. These results clearly show that health facilities are arenas of social relations that affect not only clients’ well-being^58^ but also health insurance decision making.

Health providers were divided on the genuineness of insured patients’ complaints. A District Director of Health Services’ (DDHSs) argued: “*Many more people come to the hospital and we spend time filling forms for insured patients; prolonging the time spent treating them. They don’t realise this and complain about delays.”* There were others who, though acknowledged the increased workload, admitted insured patients’ concerns. One of them said: “*These complaints are genuine. Some of us see insured patients as giving us extra work and give preference to uninsured patients.*”

Generally, clinicians are used to hurriedly writing a few words so the additional task of filling the NHIS form, psychologically drew negative reaction towards insured patients. Some were hostile to insured patients; others collected unofficial fees or demanded cash payments to avoid filling the forms.

### Shortage of Drugs on National Health Insurance Drugs List

Shortage of drugs on the NHI Drug List undermine health providers’ desire to provide quality service to insured patients. Health providers attributed the shortage to delays in claim payment which was expected to be within four weeks after claim submission to DHISs but this does not happen. A PA described the delay in payment as follows: *“Only 60% of April bill was paid in August and the remaining 40% this month [September]. I don’t know when May bill will be paid. All these make it impossible to meet patients’ drug needs.”*

Insured patients on their part, expressed their dissatisfaction of roaming looking for accredited pharmacy shops to obtain prescribed drugs covered by the NHIS which they sometimes pay for as follows:* “We go around looking for prescribed drugs which we often pay for. Since we enrolled to avoid paying money when sick, these payments discourage us from renewing our membership.”* Our survey results corroborate these complaints. About 83.7% of insured respondents and 90.3% of previously insured agreed to the statement that availability of drugs at health facilities should be improved (Table 6).

These results illustrate a significant misunderstanding regarding what insured patients, health providers and policy makers thought would stimulate enrolment and retain members. Health facilities anticipated prompt payment of claims to enable them meet insured patients’ drug needs. Insured patients expect to receive all prescribed drugs at health facilities while policy-makers thought accredited shops could augment drug shortages at health facilities.

Provision of drugs has been established to be critical in the appreciation of service delivery. Van der Geest et al write: *“Medical practitioners see pharmaceuticals as indispensable in their encounter with sick people... patients and their relatives expect medicines to solve their problems.”*^59^ This study also shows that drugs are a critical component of quality service and its shortage in health facilities increased dissatisfaction among insured patients. This contradicts policy makers’ assumption that the NHIS will improve quality of healthcare and indicate that SHISs do not automatically lead to quality service. This study thus supports the finding that health insurance has weak or no effect on quality of service^60^ and that patients’ satisfaction about the quality of care determines the degree of participation in health insurance.^61^

## Politics and Enrolment in the National Health Insurance Scheme

Social health insurance generates fierce political debates at international, national and local levels. In Ghana, the NHIS’s political stake was very high during policy making and the initial stage of implementation.^62^ Though, the political furor disappeared from public discourse after its introduction, it still persists in subtle ways among national level stakeholders. Participants who were NPP (the party in government) and NDC (the main opposition party) sympathisers mentioned that their decision to enrol or not to enrol was influenced by the politics that surrounded NHIS’ introduction but this changed later. An NDC supporter said*:*

*“I didn’t register when insurance was introduced because of politics. The NPP said all kinds of things about NDC who first brought the idea. Though NDC could not implement health insurance, they should credit them for introducing the idea.”*

One of the NPP sympathisers also explained why people were not enrolling in the NHIS as follows*: “Don’t mind NDC supporters they thought that the NHIS will die when a new government comes to power. Now they are registering because it benefits them.”* Nobody mentioned that he did not have a valid NHIS card because of politics.

However, national level key informants (staff of the MoH and GHS) were concerned that politics is undermining the effective collaboration needed to develop efficient systems to improve quality of service, stimulate enrolment and retain people in the NHIS. A GHS staff reacted to community members’ and health providers’ complaints as follows: *“These complaints are true. It is because we [MoH, GHS and the NHIA] are not meeting to develop systems to improve service delivery.”* The MoH staff I spoke with confirmed health providers’ complaints and argued that:

*“The biggest challenge we face is that the NHIA doesn’t share information. Even now that the politics that surrounded NHIS’ introduction is over, they still do not effectively engage us [MoH and GHS] to build systems to improve service delivery. They take decsions and inform us later.”*

The NHIA staff also responded to these complaints as follows:

*“The NHIS is pro-poor. The premiums is low enough for all Ghanaians to enrol. Exemptions are also provided. But, health providers’ corrupt practices result in delays in claims reimbursement and shortage of drugs. Enrolment could be improved if facilities help in the efficient management of their stock levels and service providers improve their attitudes towards insured patients.”*

Responding to the question why the NHIA does not share information and meet regularly with the MoH and DHS officials, Another staff replied:* “They read politics into whatever we do. We inform them about what we do. They delay payment of claims is to ensure that claims are thoroughly checked.”*

A critical analysis of these comments and previous studies reveal that the NHIS is highly centralised; involvement of stakeholders and technocrats in the implementation is minimal.^7,60^ and the checks and balances needed to address gaps was often missing. As Brinkerhoff observes after analysing the US health system, effective engagement of stakeholders ensures accountability and helps reveal gaps that require intervention to improve service delivery^63^ However, in the case of Ghana, effective collaboration among stakeholders to ensure independent decision making and development of efficient systems is often sacrificed for political actors control of the NHIS.

Also, the politics of the NHIS came up four years into NHIS’ implementation when the opposition NDC also made two pledges in its manifesto for the next general elections in 2008 as follows:

“*Our universal health insurance scheme will guarantee access to free healthcare in all public health institutions. It will not be district-specific and will allow for one-time premium payment (OTPP).”*^64,65^

Upon resumming office in January 2009, the government was able to nationalise the NHIS card and the insured now access healthcare in every accredited facility irrespective of where they registered. The OTPP which means that individuals will pay premium once in their life time implies the elimination of annual premium, was fiercely debated and its implications for NHIS’ long term financial sustainability questioned. The government on his part argued that the high cost of premium collection from the informal sector can be surmounted and access expanded. Abiiro and Mcintyre observe:

*“Though the OTPP potentially can lead to increases in NHIS coverage, especially within the informal sector... sustainability will largely depend on how it is designed.... The government and the policy drivers need to... examine its feasibility and long-term sustainability within the current Ghanaian economic context.”^66^*

The considerable controversy generated about OTPP’s implication for NHIS’ long term sustainability gradually disappeared from public discourse possibly due to lack of a policy document in the public domain to justify its contribution to achieving NHIS’ goals.

## Limitation of the Study

The study covered two regions in Southern Ghana. Since the socio-economic and cultural differences between the South and the North might affect health insurance decision making differently, our conclusions should be interpreted with caution.

## Conclusion

The study revealed that the NHIS’ implementation arena is littered with multi-dimensional factors located at multiple levels. People enrolled because of NHIS’ benefits and positive health provider-patient interaction. Apart from the core poor and poor households with many dependants, poverty was not a reason for not enrolling in the NHIS and renewing membership, instead, the negative influence of traditional risk-sharing arrangements, corruption, shortage of drugs, and politics are the serious challenges that need to be addressed. We thus suggest the following interventions to improve enrolment and retention rates in the NHIS.

The evidence that insurance reduces medical complications, but inundated with corruption, requires the establishment of a national health system free of cash transaction and all residents in Ghana compelled to belong to a health insurance scheme. The compulsory enrolment would be acceptable if the NHIS resonates with quality care and makes it easier and faster for the insured to access healthcare and not the reverse. This requires that the NHIA engage local and national stakeholders to create systems that improve service delivery, prompt payment of claims to enable health facilities meet insured patients’ drug requirements and stop corrupt practices. Also, the government should resource DHISs adequately to enable them deliver NHIS cards promptly to make NHIS attractive to both the rich and the poor. Above all, the politics that erupts after every election should be stopped by appointing NHIA’s chief executive as a public servant and not a political appointee who leaves office when there is a change in government. These measures could move NHIS towards the universal coverage that was announced in 2004.

Finally, we propose more qualitative study to explore further the effect of traditional mutual support arrangements and shortage of drugs on enrolment and retention in health insurance.

## Ethical issues

The research protocol was approved by the Ghana Health Service Ethic Committee (ID No. GHS-ERC: 12-1-09). Informed consent was obtained from all participants. Anonymity and confidentiality were guaranteed.

## Competing interests

Authors declare that they have no competing interests.

## Authors’ contributions

AMK, GCA, and SVG were involved in the conceptualisation and study design. The quantitative data collection and analysis was done by AMK and GCA. AMK was solely responsible for the qualitative data collection and its analysis and wrote the first draft paper. The other authors provided critical comments during the analysis and the interpretation of the data and revising the earlier draft. All authors read and approved the final manuscript.

## Authors’ affiliations

^1^School of Public Health, University of Ghana, Legon, Ghana. ^2^Department of Sociology and Anthropology, University of Amsterdam, Amsterdam, The Netherlands.

## Endnotes


^[1]^ User-fees refer to OOPPs for some healthcare services at the point of utilisation.

^[2]^ Cash and carry led to OOPP for full cost of drugs in public health facilities. It was a WHO and UNICEF initiative adopted by African Health Ministers in Bamako, Mali, in 1987. The policy was expected to improve drug supplies in public health facilities.

^[3]^ Social Security and National Insurance (SSNIT) is a government pension scheme in Ghana that most formal sector workers and their employers contribute to.

^[4]^ CHPS zone is a national programme of community-based care provided by resident nurses who are referred to as community health officers. CHPS, introduced in 1999, reduces geographical barriers to access to healthcare and provides basic level preventive and curative services for minor ailments at the community and household levels.

^[5]^ Etic account is a description of a phenomenon in terms of its meaning to the observing outsider.

^[6]^ Emic perspectives means describing behaviours and understandings in terms of meaningful experiences to the actor.

^[7]^ *Pataase* is an association of mostly fishermen who come together to support each other in times of economic stress. They provide financial assistance to members. Their main focus is funeral costs of members or their close relatives.

^[8]^ *Nnoboa* (support weeding) is a mutual help group. The name refers to local support networks formed by farmers to support each other on their farms. In these groups, trust and commitment to achieve individual and group objectives is the key principle.


## Key messages

**Implications for policy makers**

- Despite the fact that the National Health Insurance Scheme (NHIS) has increased access to healthcare, it has not eliminated financial barrier to accessing services.
- Corrupt practices especially extra payment at health facilities and payment for drugs persist.
- Attaining universal coverage require appropriate interventions to address the issues of delay in claim payment and shortage of drugs.
- The National Health Insurance Authority (NHIA) should effectively engage national and local stakeholders to create systems to improve service delivery and ensure prompt payment of claims to enable health facilities meet insured patients’ drug needs.
- The government should resource district health insurance schemes (DHISs) adequately to enable them deliver NHIS cards promptly.

**Implications for the public**

The National Health Insurance Scheme (NHIS) is an effective means to increase healthcare access in low- and middle-income countries including Ghana but effective implementation to ensure the poor, the target of the scheme, benefit is needed. Exemption should be expanded to include poor households with large membership. Also, healthcare service delivery should be improved and the NHIS made attractive to both the rich and the poor. Intensive education is also needed to make people appreciate the operational principles of the NHIS and develop positive attitudes that will move the scheme towards universal coverage. Though social health insurance generates fierce political debates, our results show that Ghanaians are putting politics aside to enrol in the scheme. However, the politics that erupts after every election need to be stopped by appointing the chief executive as a public servant and not a political appointee who should leave office with a change in government.
